# Assessment of the GLIDE Score for Prediction of Mild Tricuspid Regurgitation following Tricuspid Transcatheter Edge-to-Edge Repair

**DOI:** 10.1016/j.jacadv.2024.101523

**Published:** 2025-02-26

**Authors:** Felix Rudolph, Akhil Narang, Maria I. Körber, Kai P. Friedrichs, Johannes Kirchner, Maria Ivannikova, Paul Cremer, Peter Luedike, Tanja K. Rudolph, Tobias Geisler, Tienush Rassaf, Roman Pﬁster, Fabien Praz, Volker Rudolph, Charles J. Davidson, Mohammad Kassar, Muhammed Gerçek

**Affiliations:** a Clinic for General and Interventional Cardiology/Angiology, Herz- und Diabeteszentrum NRW, Ruhr-Universität Bochum, Bad Oeynhausen, Germany; b Clinic for General and Interventional Cardiology/Angiology, Herz- und Diabeteszentrum NRW, Med. Fakultät OWL (Universität Bielefeld), Bad Oeynhausen, Germany; c Northwestern University Feinberg School of Medicine, Chicago, Illinois, USA; d Department for Internal Medicine III, Faculty of Medicine and University Hospital Cologne, University of Cologne, Cologne, Germany; e Department of Cardiology and Vascular Medicine, West German Heart and Vascular Center, University Hospital Essen, University of Duisburg-Essen, Essen, Germany; f Department of Cardiology and Angiology, University Hospital Tübingen, Eberhard Karls Universität Tübingen, Tübingen, Germany; g Department of Cardiology, Inselspital, Bern, Switzerland; h Graduate School for Health Sciences, University of Bern, Switzerland

**Keywords:** GLIDE score, mild tricuspid regurgitation, procedural success, transcatheter edge-to-edge repair, transcatheter therapy

## Abstract

**Background:**

The GLIDE Score is an anatomical scoring system designed to predict moderate residual tricuspid regurgitation (TR) immediately following transcatheter tricuspid edge-to-edge repair (T-TEER).

**Objectives:**

The purpose of this study was to evaluate the GLIDE Score's predictive capability for achieving a postprocedural TR grade of mild or better.

**Methods:**

This retrospective analysis included 336 patients from a multicenter registry who underwent T-TEER between January 2017 and November 2022. Anatomical features were assessed using transesophageal echocardiography to calculate the GLIDE Score, which ranges from 0 to 5. The primary endpoint was a postprocedural TR grade of mild or better, assessed via periprocedural imaging. Outcomes were compared between patients with GLIDE Scores of 0 to 1 and those with scores ≥2 using logistic regression and ROC curve analysis.

**Results:**

Median age was 81 years, with no significant differences in BMI, EuroScore II, or NYHA Class across GLIDE Score cohorts. The GLIDE Score ≥2 cohort had a larger median RV basal diameter (48 mm vs 45 mm, *P* < 0.001) and more torrential TR cases (35.9% vs 3.1%, *P* < 0.001). Postprocedural mild TR was achieved in 74.7% of patients with a GLIDE Score of 0 to 1, versus 13.4% in the ≥2 cohort (*P* < 0.001). Ordinal regression analysis found a strong correlation between the GLIDE Score and postprocedural TR severity (coefficient = 1.41, t = 12.92), with an AUC to predict mild TR of 0.87 (95% CI: 0.83-0.90).

**Conclusions:**

The GLIDE Score is a valuable tool for predicting postprocedural TR severity in T-TEER patients, guiding patient selection and refining treatment strategies.

Relevant tricuspid regurgitation (TR), defined as moderate or higher, is highly prevalent in elderly patients and is associated with a significant symptomatic burden, high hospitalization rates, and increased mortality. Furthermore, the incidence of TR increases with age, posing a future challenge to health systems.^1^^,^^2^ Most cases are secondary TR, caused by the enlargement of the right atrium or right ventricle (RV) with consequent dilation of the tricuspid annulus due to underlying cardiac conditions.^3^ In recent years, the understanding of TR has shifted from being a mere side effect of these underlying diseases to being recognized as a prognostically relevant condition in its own right.^2^ However, treatment options for TR have often been limited to symptomatic relief, as surgical repair or replacement carries high periprocedural risks, particularly in elderly patients.^4^

To address this issue, transcatheter approaches have been developed. The most commonly employed treatment modality for TR is tricuspid transcatheter edge-to-edge therapy (T-TEER). Although a recent randomized controlled trial did not demonstrate a prognostic benefit of T-TEER over optimal medical therapy (OMT) alone, it did show a significant improvement in quality of life, a finding supported by additional recent publications.^5^^,^^6^ This improvement in functional status is significantly influenced by procedural success, which is commonly defined as an absolute TR reduction by 2 or more grades or to a grade of moderate or better. However, predicting procedural success prior to intervention is challenging, complicating the selection of appropriate patients for T-TEER, especially as other treatment strategies, such as transcatheter tricuspid valve replacement (TTVR), are emerging.

To address this challenge, the GLIDE Score (Gap, Location, Image quality, Density, En-face TR morphology) was proposed as the first scoring system based on anatomical features of the tricuspid valve to predict postprocedural TR grade in patients undergoing T-TEER for tricuspid regurgitation.^7^ The score was validated to predict an immediate postprocedural result of a TR grade of moderate or better or a reduction by 2 or more grades.

A matter of current debate is whether defining technical success as a reduction to a TR grade of moderate or better is sufficient or if adjusted grading methods for postprocedural results aiming for lower TR grades might better predict patient prognosis.^8^ A recent publication found that within patients classified with moderate residual TR, further subdivision in “mild-to-moderate” and “moderate-to-severe” significantly discriminates survival rates at 2 year follow-up.^8^ Therefore, the aim of this study was to analyze the capabilities of the GLIDE Score in predicting an immediate postprocedural TR reduction to a mild or better grade.

## Methods

### Study design

This study is a retrospective analysis of an international multicenter registry for all adult patients who underwent T-TEER and who provided written consent to participate in the study at 5 tertiary centers, 4 in Germany and 1 in Switzerland, between January 2017 and November 2022. All patients underwent transthoracic and transesophageal (TEE) assessment prior to intervention. Severity of TR was graded in 5 grades from mild to torrential. An interdisciplinary heart team on site decided the employed treatment modality; the implanted device was chosen by the interventionalist. Primary endpoint was a TR grade of mild or less at the end of the procedure as quantified via periprocedural TEE, as venca contracta <3 mm or effective regurgitant orifice area <20 mm^2^. Technical success was defined as successful device deployment without detachment, retrieval of the device delivery system without the need for emergency surgery, and the patient being alive at the end of the procedure. Device success was defined according to the Tricuspid Valve Academic Research Consortium criteria as: absence of intraprocedural mortality or stroke, successful access, delivery, and retrieval of the device delivery system, successful deployment and correct positioning of the intended device(s) without requiring implantation of unplanned additional devices, absence of tricuspid stenosis and a reduction of total tricuspid regurgitation to optimal moderate or better, absence of device-related obstruction of forward flow, device-related pulmonary embolism, and freedom from emergency surgery or reintervention during the first 24 h related to the device or access procedure.^9^ Data collection was approved by the local Institutional Review Boards and conducted in accordance with local data protection regulations. Written informed consent was obtained from every patient.

### GLIDE score

The GLIDE Score incorporates 5 anatomical features derived from TEE. For each feature, either no or one point is given based on complexity (see Table 1). Accordingly, the score ranges from 0 to a maximum of 5 points with more points translating to more complex anatomies and lower chance of optimal postprocedural results. Quantification of the GLIDE Score for each patient was conducted by a core laboratory blinded to procedural results based on preprocedural TEE. For group comparisons, patients were divided into 2 cohorts: those with a GLIDE Score of 0 or 1 and those with a GLIDE Score of ≥2, as this division demonstrated good discrimination within the original study.^7^

**Table 1:** The GLIDE Scoring System^7^

	Parameters	Straightforward	Complex
		0 points	1 point
G	Septolateral gap	0-5 mm	>5 mm
L	Predominant jet location	Anteroseptal or central	Posteroseptal or anterioposterior or diffuse
I	Image quality	Good	Limited
D	Chordal structure density	Modest	High
E	En-Face TR jet morphology	Oval or linear	Star-Shaped

TR = tricuspid regurgitation.

### Statistical analysis

Statistical analyses were performed using R in RStudio (Version 2023.06, RStudio Inc). Continuous variables were reported as mean ± SD if normally distributed and as median (IQR) if not normally distributed. Categorical variables were presented as frequencies and percentages. The Shapiro–Wilk test was used to determine if the data were normally distributed. Group comparisons for continuous variables were conducted using the Wilcoxon test or Student’s t-test, as appropriate. Categorical group comparisons were calculated using Fisher’s exact test. Correlations between GLIDE Scores and postprocedural TR grades were assessed using ordinal regression analysis. The predictive capability of the GLIDE Score for the postprocedural TR grade of mild was assessed using logistic regression analysis. The goodness-of-fit of the logistic regression model was assessed using the Hosmer–Lemeshow test. Additionally, a likelihood ratio test was performed to compare the full model with an intercept-only model. Receiver operating characteristic analysis was conducted employing the “pROC” package (V 1.18.5) in R. A *P* value <0.05 was considered statistically significant. Diagnostic performance metrics, including sensitivity, specificity, positive predictive value, and negative predictive value, were calculated to evaluate the predictive capability of the GLIDE Score.

## Results

### Study population

A total of 336 patients were included in the analysis. Of these, 168 were from the original derivation cohort used to create the GLIDE Score. Based on the GLIDE Score, patients were divided in 2 cohorts: 194 patients with a GLIDE Score of 0 to 1 and 142 patients with a GLIDE Score of ≥2. The median age was 81 (IQR: 77-84) years, with a similar proportion of male patients (GLIDE 0-1: 42.8% vs GLIDE ≥2: 47.2%, *P* = 0.40). No significant differences in body mass index, EuroScore II, or relevant comorbidities were found between the cohorts. The NYHA functional class was ≥ III in 146 (55.9%) of all patients without a significant difference between the cohorts (*P* = 0.08). While the Tricuspid Annular Systolic Excursion and RV fractional area change were comparable between the cohorts, the RV basal diameter was significantly higher in the GLIDE Score ≥2 cohort (GLIDE 0-1: 45 [IQR: 40-50] mm vs GLIDE ≥2: 49 [IQR: 43-54] mm, *P* < 0.001). Notably, NT-proBNP levels and pulmonary hemodynamic parameters did not differ significantly between the cohorts. However, TR etiology and severity revealed variations: the GLIDE Score ≥2 cohort had a higher percentage of patients with torrential TR (GLIDE 0-1: 3.1% vs GLIDE ≥2: 35.9%, *P* < 0.001). The baseline characteristics are summarized in Table 2.

**Table 2:** Baseline Characteristics

	Availability	All (N = 336)	GLIDE Score 0-1	GLIDE Score ≥2	*P* Value
			(n = 194)	(n = 142)	
Age, y	336/336	81 (77-84)	81 (77-84)	81 (76-84)	0.56
Male	336/336	150 (44.6)	83 (42.8)	67 (47.2)	0.44
BMI, kg/m^2^	336/336	25.2 (22.7-28.4)	25.3 (22.6-28.7)	25.1 (22.9-28.1)	0.80
EuroScore II, %	336/336	5.2 (3.3-9.1)	4.8 (3.3-8.5)	5.5 (3.4-10.9)	0.25
NYHA functional class ≥III	261/336	146 (55.9)	76 (48.7)	70 (66.7)	0.08
Atrial fibrillation	336/336	306 (91.3)	177 (91.2)	129 (91.5)	>0.99
Diabetes	336/336	80 (23.8)	48 (24.7)	32 (22.5)	0.70
COPD	336/336	57 (17.0)	29 (15.0)	28 (19.7)	0.30
CAD	336/336	161 (47.9)	95 (49.0)	66 (46.5)	0.70
History of cardiac surgery	336/336	102 (30.4)	51 (26.3)	51 (35.9)	0.07
History of stroke	293/336	44 (15.0)	26 (14.9)	18 (15.1)	0.87
Renal failure requiring dialysis	336/336	12 (3.6)	5 (2.6)	7 (4.9)	0.37
NT-proBNP, pg/mL	306/336	2,695 (1,427-5,228)	2,620 (1,422-4,730)	2,744 (1,502-6,105)	0.30
mPAP, mm Hg	263/336	28 (23-35)	29 (23-34)	28 (24-35)	0.60
PCWP, mm Hg	250/336	19 (14-24)	18 (14-23)	20 (15-26)	0.10
PVR, mm Hg	211/336	3 (2-4)	3 (2-4)	3 (2-4)	0.38
LVEF, %	331/336	55 (48-63)	56 (47-63)	55 (50-63)	0.70
TAPSE, mm	297/336	17 (14-20)	17 (14-20)	17 (14-19)	0.40
RV basal diameter, mm	326/336	46 (41-53)	45 (40-50)	48 (43-54)	<0.001
RV-FAC, %	326/336	40 ± 11	41 ± 11	40 ± 10	0.30
Degenerative TR	294/336	12 (4.1)	7 (4.0)	5 (4.2)	>0.99
Atrial TR	294/336	138 (46.9)	89 (51.2)	49 (40.8)	0.043
Ventricular TR	294/336	144 (49.0)	78 (44.8)	66 (55.0)	0.27
Severe TR	336/336	164 (48.8)	133 (68.6)	31 (21.8)	<0.001
Massive TR	336/336	115 (34.2)	55 (28.4)	60 (42.3)	0.010
Torrential TR	336/336	57 (17.0)	6 (3.1)	51 (35.9)	<0.001

Values are n/N, median (IQR), or n (%).

BMI = body mass index; CAD = coronary artery disease; COPD = chronic obstructive pulmonary disease; LVEF = left-ventricular ejection fraction; mPAP = mean pulmonary artery pressure; PCWP = pulmonary capillary wedge pressure; PVR = pulmonary vascular resistance; RV = right ventricle; RV-FAC = right-ventricular fractional area change; TAPSE = tricuspid annular systolic excursion; TR = tricuspid regurgitation.

### Procedure details

The median number of implanted devices was 2 (IQR: 1-2). Procedure time was 89 (IQR: 66-116) min in the GLIDE Score 0 to 1 cohort and 125 (IQR: 87-158) min in the GLIDE Score ≥2 patients (*P* < 0.001). Rates of achieved device and technical success were significantly higher in the GLIDE Score 0 to 1 cohort at 86.8% versus 43.3% (*P* < 0.001) and 98.3% versus 89.2% (*P* = 0.003), respectively (see Table 3). Procedure-related complications revealed no significant differences between the groups (see Supplemental Table 1). The TriClip system was employed in 134 (39.9%) and the Pascal system in 202 (60.1%) of all patients without significant differences for these percentages based on the GLIDE Score cohort (*P* = 0.50). Detailed clinical and procedural characteristics based on the implanted system are presented in Supplemental Tables 2 to 4.

**Table 3:** Procedural Details

	Availability	All (N = 336)	GLIDE Score 0-1	GLIDE Score ≥2	*P* Value
			(n = 194)	(n = 142)	
Procedure time, min	188/336	101 (71-132)	89 (66-116)	125 (87-158)	<0.001
TriClip System employed	336/336	134 (39.9)	74 (38.1)	60 (42.3)	0.50
Pascal System employed	336/336	202 (60.1)	120 (61.9)	82 (57.8)	0.50
Device success	294/336	203 (69.1)	151 (86.8)	52 (43.3)	<0.001
Technical success	294/336	278 (94.6)	171 (98.3)	107 (89.2)	0.003
Mild TR achieved	336/336	164 (48.8)	145 (74.8)	19 (13.4)	<0.001

Values are n/N, median (IQR), or n (%).

TR = tricuspid regurgitation.

### GLIDE score

The median GLIDE Score for all patients was 1 (IQR: 0-3), with 194 (57.7%) of patients scoring 0 or 1 point and 142 (42.3%) scoring ≥2 points. Of all patients, 32 (9.5%) scored ≥4 points. Predominant jet location was the most common criterion, noted in 50.6% of all patients. It was more frequent in the GLIDE Score ≥2 cohort at 84.5%, compared to 25.8% in the GLIDE Score 0 to 1 cohort (*P* < 0.001). En-face TR jet morphology was the second most common criterion, observed in 39.6% of all patients, and was noted more often in the GLIDE Score ≥2 cohort at 81.7% versus 8.8% in the GLIDE Score 0 to 1 patients (*P* < 0.001). Image quality was the least fulfilled criterion, seen in 18.8% of all patients. Significant differences were observed between the cohorts for all 5 criteria (see Table 4). The GLIDE Score correlated significantly with the severity of TR at baseline (spearman’s rho = 0.53, *P* < 0.001).

**Table 4:** Composition of GLIDE Score Results

	Availability	All (N = 336)	GLIDE Score 0-1	GLIDE Score ≥2	*P* Value
			(n = 194)	(n = 142)	
GLIDE Score sum	336/336	1 (0-3)	1 (0-1)	3 (2-3)	<0.001
Septolateral gap	336/336	77 (22.9)	3 (1.5)	74 (52.1)	<0.001
Predominant jet location	336/336	170 (50.6)	50 (25.8)	120 (84.5)	<0.001
Image quality	336/336	63 (18.8)	22 (11.3)	41 (28.9)	0.027
Chordal structures density	336/336	69 (20.5)	10 (5.2)	59 (41.5)	<0.001
En-Face TR jet morphology	336/336	133 (39.6)	17 (8.8)	116 (81.7)	<0.001

Values are n/N, median (IQR), or n (%).

TR = tricuspid regurgitation.

### Prediction of postprocedural mild TR

**Figure 1:**
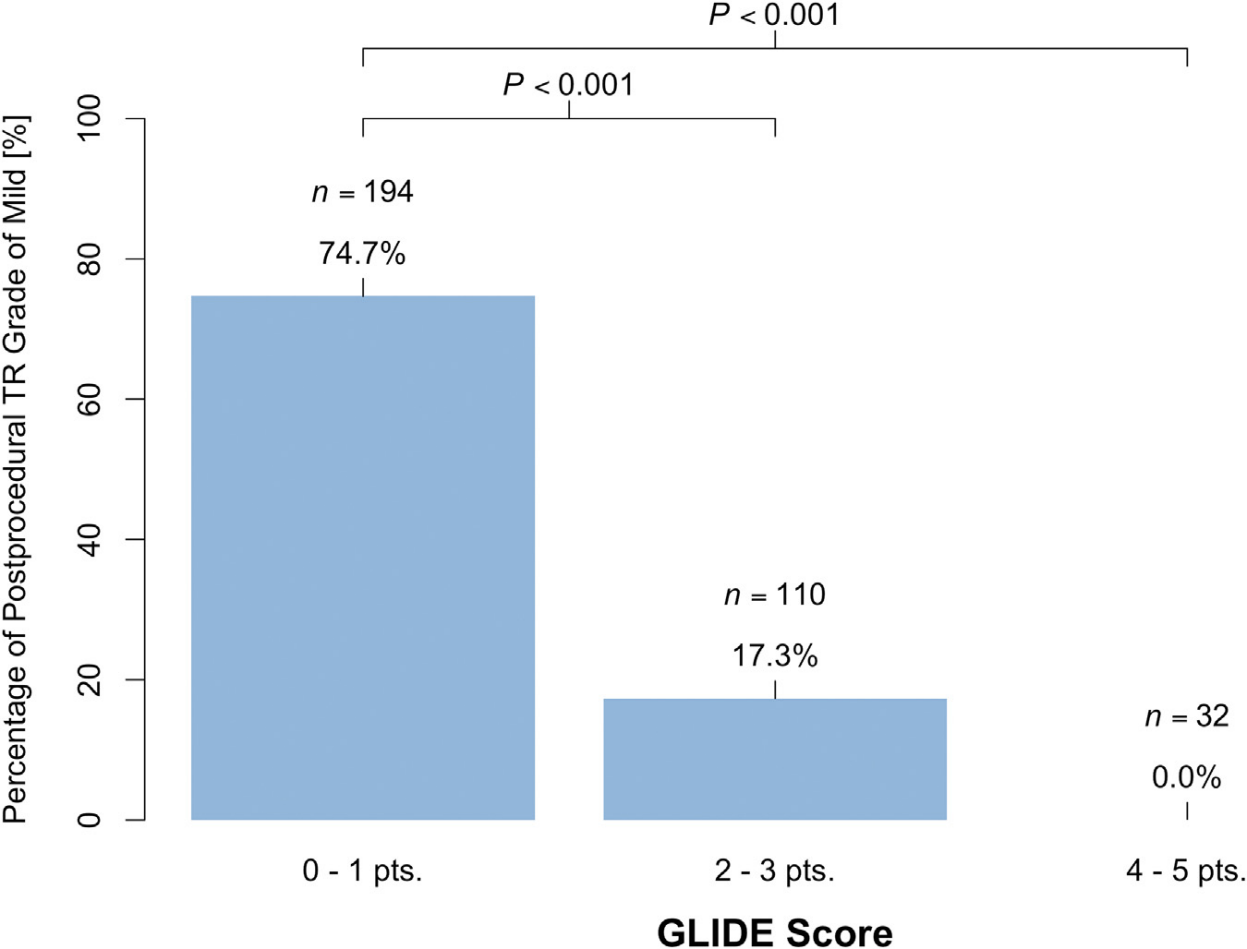
**Percentage of Postprocedural TR Grade of Mild Depending on GLIDE Score** The GLIDE Score was assessed based on anatomical features derived from preprocedural transesophageal echocardiography in 336 patients. pts. = points; TR = tricuspid regurgitation.

**Figure 2:**
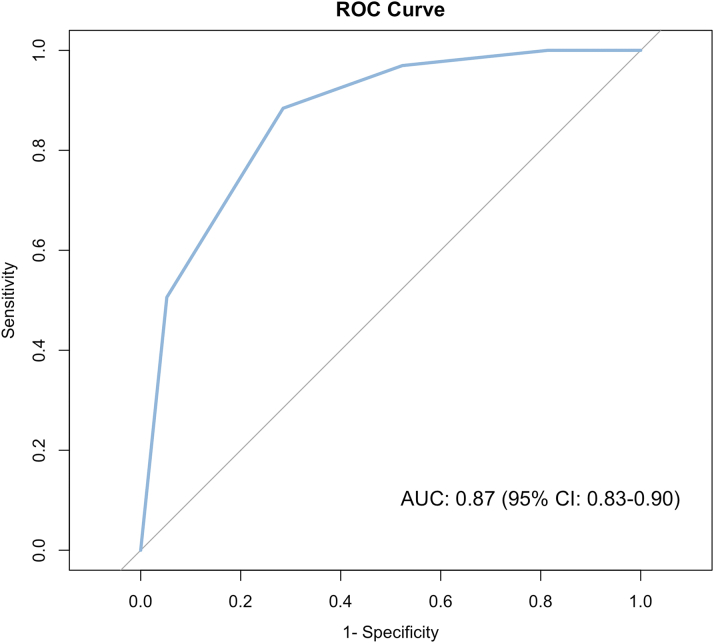
**ROC Curve for the GLIDE Score to Predict Mild TR after T-TEER** AUC = area under the curve.

The primary endpoint of a TR grade of mild or less was achieved in 74.7% of patients in the GLIDE Score 0 to 1 cohort, while only 17.3% of the patients in the GLIDE Score ≥2 cohort reached this endpoint (*P* < 0.001). None of the patients classified with a GLIDE Score ≥4 reached this endpoint (Figure 1). Ordinal regression analysis revealed a significant positive correlation (coefficient = 1.41, t = 12.92), indicating a strong association between higher GLIDE Scores and more severe postprocedural TR grades. Logistic binary regression revealed a negative association between GLIDE Score sum and achievement of the primary endpoint (OR: 0.21 (95% CI: 0.15-0.28), *P* < 0.001). The Hosmer–Lemeshow goodness-of-fit test indicated no significant difference between observed and expected event rates (chi-squared = 0.50, *P*-value = 0.78) and the likelihood ratio test comparing the intercept-only model and the model including the GLIDE Score as the predictor showed a significant reduction in deviance (chi-square = 172.62, *P* < 0.001). We tested different cut-offs for the primary outcome of mild residual TR for best discrimination. A cut-off of 2 showed the best discrimination with a sensitivity of 0.88 (95% CI: 0.83-0.93), specificity of 0.72 (95% CI: 0.64-0.78), positive predictive value of 0.75 (95% CI: 0.68-0.80), and negative predictive value of 0.87 (95% CI: 0.80-0.91). The area under the curve was calculated to be 0.87 (95% CI: 0.83-0.90) (see Figure 2). Subgroup analysis based on the implanted TEER system (TriClip or Pascal) showed comparable capabilities of the GLIDE Score to predict mild residual TR (see Supplemental Table 5).

## Discussion

In this analysis of 336 T-TEER patients from an international multicenter registry, we found that the GLIDE Score was predictive of achieving a postprocedural TR grade of mild. The present analysis expands on the findings of the original study, in which the GLIDE Score was initially described and validated by: 1) incorporating further patients from an additional tertiary center; and 2) validating the predictive capabilities for mild residual TR. We found the GLIDE Score to be both predictive of ordinal postprocedural TR grade, as well as binary for achievement of mild TR or not. For the latter, an area under the curve of 0.87 was found with an optimal threshold of 2.2. Among patients with a GLIDE Score of 0 to 1, 74.7% achieved an immediate postprocedural TR grade of mild. This proportion decreased to 17.3% for those with a GLIDE Score of 2 to 3, and no patient with a GLIDE Score of 4 or higher achieved a TR grade of mild (see Central Illustration). Although this study did not systematically record data on patients excluded from T-TEER, a previous analysis of the anatomical characteristics of screen-failure patients for transcatheter tricuspid valve repair found that the majority were excluded due to severe enlargement of tricuspid valve annulus, right atrium, and RV.^10^

**Central Illustration:**
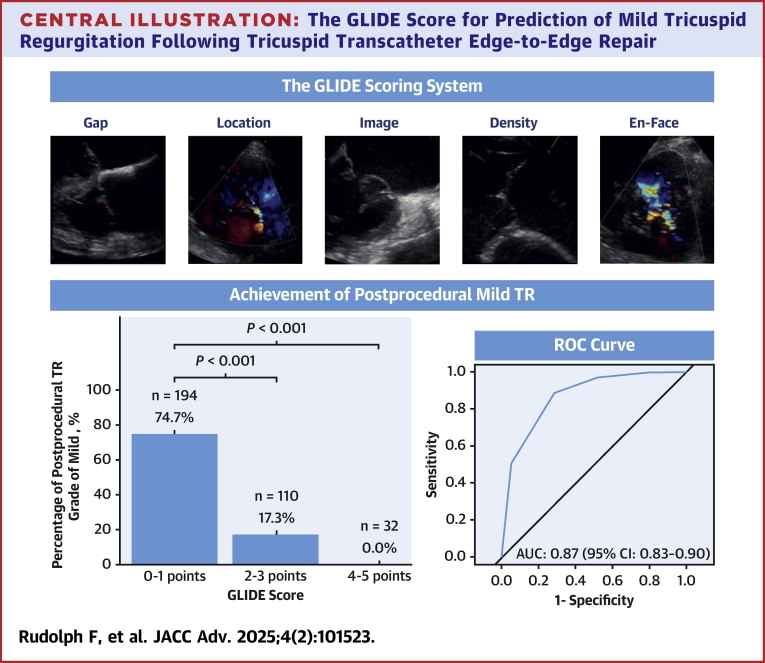
**The GLIDE Score for Prediction of Mild Tricuspid Regurgitation Following Tricuspid Transcatheter Edge-to-Edge Repair** TR = tricuspid regurgitation.

Dreyfus et al^8^ recently published data from a subanalysis of the TRIGISTRY, examining the impact of residual TR after transcatheter tricuspid valve repair in 613 patients (492 T-TEER and 115 direct annuloplasty). Survival rates at 2 years decreased with higher residual TR grades.^8^ Survival rates within those patients classified as moderate were strongly heterogenous, hence the proposal to further discriminate this grade into “mild-to-moderate” and “moderate-to-severe”, effectively proposing a 6-grade scheme for tricuspid regurgitation. The exact criteria employed to discriminate between these 2 newly introduced grades were not mentioned, other than being similar to the grading scheme applied to edge-to-edge therapy for mitral regurgitation. The need for further validation of this proposed new grading scheme is acknowledged by the authors.^8^ For the current scheme, no significant differences were found for survival rates when comparing mild to moderate TR at discharge in the analyzed data from TRIGISTRY.^8^ This is consistent with data from EuroTR, where a significant difference in survival was found for moderate versus severe residual TR, but not between mild and moderate.^11^ Interestingly, the Kaplan–Meier curves for mild and moderate residual TR from the TRIGISTRY and EuroTR registries appear to diverge significantly earlier in the smaller TRIGISTRY dataset, raising concerns about the applicability of the study results to the entirety of performed procedures.^8^^,^^11^

Another aspect is whether the increased mortality with higher residual TR is causative of the residual TR or associative with underlying advanced disease, possibly associated with left-sided cardiac disease as a confounder of increased mortality. Although the authors introduced risk adjustment and tested multiple multivariate models, this possible impact of confounding factors needs to be considered, especially in patient collectives treated with T-TEER.

It seems intuitive that lower grades of residual TR are associated with better survival rates, as reflected in data available for conservatively treated TR.^12^ Within these data for patients with heart failure with reduced ejection fraction and functional TR, significantly worse survival rates were found with increasing TR grades under conservative management after 10 years.^12^ However, data on the impact of residual TR after transcatheter therapy is still scarce. The TRILUMINATE pivotal trial failed to provide evidence for increased prognosis when compared T-TEER to OMT alone but found improved functional status in the patients treated with T-TEER plus OMT.^6^ This improvement was more prevalent in patients in which a residual TR grade of moderate or better was achieved.^5^^,^^6^

The effort to achieve the least amount of residual TR should be the goal in every procedure performed. However, if the proposed grading scheme by Dreyfus et al^8^ actually provides a benefit in clinical risk assessment remains to be seen, especially as this introduces more complexity to the echocardiographic assessment at follow-up. Furthermore, this focus on residual TR grade to identify suboptimal results prone to poorer prognosis neglects assessment by the treating team of physicians based on clinical appearance and comorbidities. Ideally, risk assessment would be conducted prior to intervention when other strategies might still be considered, not following costly implantation of devices possibly limiting further interventions such as TTVR. In the present analysis, none of the patients with a GLIDE score of 4 points or higher reached the endpoint of mild residual TR. Given the current debate about whether achieving this endpoint represents a desirable goal for tricuspid valve intervention with prognostic benefit compared to moderate residual TR, it might be concluded that T-TEER should not be performed in these patients. While conditions for T-TEER might be optimized by managing optimal volume status prior to an intervention, other treatment strategies such as direct annuloplasty or TTVR should be evaluated in such advanced cases.

The GLIDE Score is the first readily available tool for predicting procedural results and can aid in these decisions. A limitation of the score is that it is validated only for immediate postprocedural results, which may be influenced by volume status and sedatives used during the procedure. These factors might introduce variations between the immediate postprocedural results and TR grades at discharge or follow-up. However, the postprocedural result has been shown to correlate to improvements in functional status after 30 days.^7^

While currently no prognostic benefit of residual mild TR was proven over moderate, the study by Dreyfus et al^8^ indicates an association between lower residual TR grades with improved survival rates. Hence, we tested the GLIDE Score to predict mild residual TR immediately following T-TEER and found excellent predictive capabilities. While this analysis did not consider the proposed expanded grading scheme, as the GLIDE Score, originally created to predict moderate TR, was now also proven to be predictive of mild residual TR. Hence, if the proposed further differentiation of moderate TR is adopted in routine practice, the GLIDE Score should also be able to predict “mild-to-moderate” TR, which might be associated with better survival. GLIDE Scores capabilities to predict long-term outcomes, both in terms of TR grade and functional status, is a matter of further investigation.

### Study Limitations

This analysis has several limitations. First, the study was conducted in a retrospective manner including patients treated up until November 2022. Advances in device technology, patient selection, and clinical experience since then may affect the generalizability of our findings. Second, the GLIDE Score has only been validated for immediate postprocedural outcomes. Although these have been shown to correlate with improvements in functional status at 30 days, its effectiveness in predicting long-term TR reduction remains to be assessed. Third, while recent discussions have focused on the introduction of an advanced TR grading scheme, our study evaluated the GLIDE Score based solely on the current grading system. Further research is necessary to explore the score’s utility with the proposed advanced grading scheme. Fourth, the GLIDE Score was developed based on retrospective data of patients who had already undergone T-TEER. Thus, a selection bias favoring T-TEER interventions cannot be fully excluded. Fifth, the criterion of chordal structure density might be prone to interobserver variability when assessed in a nonstandardized manner.

## Conclusions

The impact of distinguishing between mild and moderate residual TR following T-TEER on patient prognosis remains debated. Recent proposals to refine TR grading by dividing moderate TR into mild-to-moderate and moderate-to-severe categories aim to enhance the prediction of patient outcomes. Identifying patients with a higher risk of poor prognosis prior to treatment is crucial. In this study, we evaluated the GLIDE Score for its ability to predict postprocedural mild TR and found an excellent predictive performance. These findings suggest that the GLIDE Score is effective in predicting a postprocedural TR grade of mild or less in patients undergoing T-TEER for TR.

Perspectives **COMPETENCY IN PATIENT CARE AND PROCEDURAL SKILLS:** The ability of the GLIDE Score to effectively predict postprocedural mild TR may have prognostic implications when compared to residual moderate TR. This predictive capability allows clinicians to better identify patients who are more likely to achieve favorable outcomes following T-TEER. By integrating the GLIDE Score into clinical practice, healthcare providers can enhance patient selection and personalize therapeutic strategies.

**TRANSLATIONAL OUTLOOK:** This targeted approach ensures that patients at higher risk of residual TR receive more appropriate and tailored interventions, potentially leading to improved clinical outcomes and more efficient resource utilization in the management of tricuspid regurgitation.

## Funding support and authors disclosures

Dr Felix Rudolph has received funding from Bielefeld University (clinician scientist entry fellowship). Dr Narang has received speaker honoraria from Edwards Lifesciences. Dr Friedrichs is consultant for and has received speaker honoraria from Edwards Lifesciences. Dr Ivannikova has received speaker honoraria from Edwards and AstraZeneca. Dr Luedike has received speaker honoraria and consulting fees from AstraZeneca, Bayer, Pfizer, and Edwards Lifesciences; and has received research honoraria from Edwards Lifesciences. Dr Tanja Rudolph has received speaker honoraria from Edwards Lifesciences. Dr Kassar reports research grants from the swiss and german heart foundation. Dr Rassaf has received honoraria, lecture fees, and grant support from 10.13039/100006520Edwards Lifesciences, 10.13039/100004325AstraZeneca, 10.13039/100004326Bayer, 10.13039/100004336Novartis, 10.13039/501100022535Berlin Chemie, Daiicho-Sankyo, 10.13039/100001003Boehringer Ingelheim, 10.13039/501100004191Novo Nordisk, Cardiac Dimensions, and 10.13039/100004319Pfizer, all unrelated to this work; and he is co-founder of Bimyo GmbH, a company that develops cardioprotective peptides. Dr Pfister has received honorarium for consultation from Edwards Lifescience. Dr Praz has received travel expenses from Abbott Vascular, Edwards Lifesciences, Polares Medical, and Siemens Healthineers. Dr Volker Rudolph has received grants and speaker honoraria from Abbott and Edwards Lifesciences. Dr Davidson has received grants from 10.13039/100000046Abbott and 10.13039/100006520Edwards Lifesciences; is an uncompensated consultant for Edwards Lifesciences; and has received honoraria from Philips Healthcare. Muhammed Gercek has received funding from the 10.13039/501100006254Ruhr University Bochum (advanced clinician scientist grant) and speaker honoraria from Edwards Lifesciences. All other authors have reported that they have no relationships relevant to the contents of this paper to disclose.
