# Supplementary material for: Assessment of the GLIDE Score for Prediction of Mild Tricuspid Regurgitation following Tricuspid Transcatheter Edge-to-Edge Repair
Source: JACC Adv. 2025 Feb 26;4(2):101523. doi: 10.1016/j.jacadv.2024.101523 (PMC11905155; doi:10.1016/j.jacadv.2024.101523)
Supplement: Supplemental Tables [file mmc1.docx]

**Supplemental Table 1:** Detailed Overview of Procedural Complications depending on GLIDE Score.

|  |  | **All** | **GLIDE Score**  **0 - 1** | **GLIDE Score**  **≥ 2** |  |
| --- | --- | --- | --- | --- | --- |
|  | Availability | *n* = 336 | *n* = 194 | *n* = 142 | *P*-value |
| All-cause mortality at 30 days, n (%) | 293/336 | 8 (2.7) | 3 (1.7) | 5 (4.2) | 0.29 |
| Cardiovascular mortality at 30 days, n (%) | 293/336 | 7 (2.4) | 3 (1.7) | 4 (3.3) | 0.46 |
| Bleeding requiring transfusion, n (%) | 294/336 | 15 (5.1) | 7 (4.0) | 8 (6.7) | 0.43 |
| Renal failure requiring dialysis, n (%) | 294/336 | 6 (2.0) | 5 (2.9) | 1 (0.8) | 0.41 |
| Device detatchment, n (%) | 294/336 | 11 (3.7) | 4 (2.3) | 7 (5.8) | 0.21 |
| Reintervention for tricuspid valve dysfunction at 30 days, n (%) | 292/336 | 6 (2.1) | 3 (1.7) | 3 (2.5) | 0.70 |
| Cardiac structural complications, n (%) | 294/336 | 2 (0.7) | 1 (0.6) | 1 (0.8) | >0.99 |
| Cerebrovascular event at 30 days, n (%) | 292/336 | 1 (0.3) | 1 (0.6) | 0 (0.0) | >0.99 |

**Supplemental Table 2:** Clinical and Procedural Details Based Implanted Device System.

|  |  | **All** | **TriClip** | **Pascal** |  |
| --- | --- | --- | --- | --- | --- |
|  | Availability | *n* = 336 | *n* = 134 | *n* = 202 | *P*-value |
| Age, years | 336 / 336 | 81 (77-84) | 81 (77-84) | 81 (77-84) | 0.94 |
| Male, n (%) | 336 / 336 | 150 (44.6) | 62 (46.3) | 88 (43.6) | 0.66 |
| BMI, kg/m^2^ | 336 / 336 | 25.2 (22.7-28.4) | 25.4 (22.4-28.1) | 25.2 (22.8-28.7) | 0.64 |
| EuroScore II, % | 336 / 336 | 5.2 (3.3-9.1) | 5.5 (3.5-10.3) | 4.8 (3.0-9.0) | 0.07 |
| NYHA-FC ≥ III, n (%) | 261 / 336 | 146 (55.9) | 68 (66.0) | 78 (49.4) | 0.033 |
| Atrial Fibrillation, n (%) | 336 / 336 | 306 (91.3) | 125 (93.3) | 181 (90.1) | 0.33 |
| Diabetes, n (%) | 336 / 336 | 80 (23.8) | 30 (22.4) | 50 (24.8) | 0.70 |
| COPD, n (%) | 336 / 336 | 57 (17.0) | 24 (17.9) | 33 (16.3) | 0.77 |
| CAD, n (%) | 336 / 336 | 161 (47.9) | 72 (53.7) | 89 (44.1) | 0.10 |
| History of Cardiac  Surgery, n (%) | 336 / 336 | 102 (30.4) | 45 (33.6) | 57 (28.2) | 0.33 |
| History of Stroke, n (%) | 293 / 336 | 44 (15.0) | 17 (16.7) | 27 (14.1) | >0.99 |
| Renal failure  requiring dialysis, n (%) | 336 / 336 | 12 (3.6) | 7 (5.2) | 5 (2.5) | 0.23 |
| NT-proBNP, pg/ml | 306 / 336 | 2695 (1427-5228) | 2905 (1347-5663) | 2508 (1465-4844) | 0.53 |
| mPAP, mmHg | 263 / 336 | 28 (23-35) | 29 (23-35) | 28 (23-34) | 0.96 |
| PCWP, mmHg | 250 / 336 | 19 (14-24) | 19 (14-25) | 18 (14-22) | 0.27 |
| PVR, mmHg | 211 / 336 | 3 (1.9-4.1) | 3 (1.7-4.3) | 3 (2.0-4.0) | 0.69 |
| LVEF, % | 331 / 336 | 55 (48-63) | 55 (45-62) | 57 (50-63) | 0.023 |
| TAPSE, mm | 297 / 336 | 17 (14-20) | 17 (14-19) | 17 (14-20) | 0.95 |
| RV basal diameter, mm | 326 / 336 | 46 (41.1-52.8) | 46 (41-52) | 46 (42-53) | 0.77 |
| RV-FAC, % | 326 / 336 | 40.3 ± 11.0 | 39.4 ± 10.6 | 40.9 ± 11.2 | 0.22 |
| Degenerative TR, n (%) | 294 / 336 | 12 (4.1) | 4 (3.9) | 8 (4.2) | 0.77 |
| Atrial TR, n (%) | 294 / 336 | 138 (46.9) | 53 (51.5) | 85 (44.5) | 0.65 |
| Ventricular TR, n (%) | 294 / 336 | 144 (49.0) | 46 (44.7) | 98 (51.3) | 0.013 |
| Severe TR, n (%) | 336 / 336 | 164 (48.8) | 60 (44.8) | 104 (51.5) | 0.27 |
| Massive TR, n (%) | 336 / 336 | 115 (34.2) | 48 (35.8) | 67 (33.2) | 0.64 |
| Torrential TR, n (%) | 336 / 336 | 57 (17.0) | 26 (19.4) | 31 (15.4) | 0.37 |
| Mild TR achieved, n (%) | 336 / 336 | 164 (48.8) | 61 (45.5) | 103 (51.0) | 0.37 |
| Procedure time, min | 188 / 336 | 101 (71-132) | 88 (65-120) | 107 (73-135) | 0.09 |
| Number of implanted devices, n | 336 / 336 | 2 (1-2) | 2 (1-2) | 2 (1-2) | 0.07 |
| Technical success, n (%) | 294 / 336 | 278 (94.6) | 93 (90.3) | 185 (96.9) | >0.99 |
| Device success, n (%) | 294 / 336 | 203 (69.1) | 67 (65.1) | 136 (71.2) | 0.002 |
| All-cause mortality at 30 days, n (%) | 293 / 336 | 8 (2.7) | 5 (4.9) | 3 (1.6) | 0.27 |
| Cardiovascular mortality at 30 days, n (%) | 293 / 336 | 7 (2.4) | 4 (3.9) | 3 (1.6) | 0.44 |
| Bleeding requiring transfusion, n (%) | 294 / 336 | 15 (5.1) | 6 (5.8) | 9 (4.7) | >0.99 |
| Renal failure requiring dialysis, n (%) | 294 / 336 | 6 (2.0) | 5 (4.9) | 1 (0.5) | 0.039 |
| Device detatchment, n (%) | 294 / 336 | 11 (3.7) | 6 (5.8) | 5 (2.6) | 0.36 |
| Reintervention for tricuspid valve dysfunction at 30 days, n (%) | 292 / 336 | 6 (2.1) | 3 (2.9) | 3 (1.6) | 0.69 |
| Cardiac structural complications, n (%) | 294 / 336 | 2 (0.7) | 1 (1.0) | 1 (0.5) | >0.99 |
| Cerebrovascular event at 30 days, n (%) | 292 / 336 | 1 (0.3) | 1 (1.0) | 0 (0.0) | 0.40 |

TR = Tricuspid Regurgitation, BMI = Body-Mass-Index, NYHA-FC = New York Heart Association Functional Class, COPD = Chronic Obstructive Pulmonary Disease, CAD = Coronary Artery Disease, mPAP = Mean Pulmonary Artery Pressure, PCWP = Pulmonary Capillary Wedge Pressure, PVR = Pulmonary Vascular Resistance, LVEF = Left-ventricular Ejection Fraction, TAPSE = Tricuspid Annular Systolic Excursion, RV = Right Ventricle, RV-FAC = Right-ventricular Fractional Area Change.

**Supplemental Table 3:** Clinical and Procedural Details Based on GLIDE Score for Patients Treated with the TriClip System.

|  |  | **All** | **GLIDE Score**  **0 - 1** | **GLIDE Score**  **≥ 2** |  |
| --- | --- | --- | --- | --- | --- |
|  | Availability | *n* = 134 | *n* = 74 | *n* = 60 | *P*-value |
| Age, years | 134/134 | 81 (77-84) | 81 (78-84) | 80 (76-83) | 0.14 |
| Male, n (%) | 134/134 | 62 (46.3) | 30 (40.5) | 32 (53.3) | 0.17 |
| BMI, kg/m^2^ | 134/134 | 25.4 (22.4-28.1) | 26.0 (22.8-28.5) | 24.2 (22.0-27.6) | 0.10 |
| EuroScore II, % | 134/134 | 5.5 (3.5-10.3) | 5.3 (3.4-8. 5) | 6.2 (4.0-12.2) | 0.19 |
| NYHA-FC ≥ III, n (%) | 103/134 | 68 (66.0) | 32 (57.1) | 36 (76.6) | 0.06 |
| Atrial Fibrillation, n (%) | 134/134 | 125 (93.3) | 68 (91.9) | 57 (95.0) | 0.73 |
| Diabetes, n (%) | 134/134 | 30 (22.4) | 16 (21.6) | 14 (23.3) | 0.84 |
| COPD, n (%) | 134/134 | 24 (17.9) | 13 (17.6) | 11 (18.3) | >0.99 |
| CAD, n (%) | 134/134 | 72 (53.7) | 40 (54.1) | 32 (53.3) | >0.99 |
| History of Cardiac  Surgery, n (%) | 134/134 | 45 (33.6) | 22 (29.7) | 23 (38.3) | 0.36 |
| History of Stroke, n (%) | 102/134 | 17 (16.7) | 12 (20.0) | 5 (11.9) | 0.20 |
| Renal failure  requiring dialysis, n (%) | 134/134 | 7 (5.2) | 2 (2.7) | 5 (8.3) | 0.24 |
| NT-proBNP, pg/ml | 124/134 | 2905 (1347-5663) | 2995 (1640-5415) | 2705 (1266-6048) | 0.80 |
| mPAP, mmHg | 110/134 | 29 (23-35) | 30 (23-37) | 29 (22-34) | 0.46 |
| PCWP, mmHg | 104/134 | 19 (14-25) | 19 (14-25) | 19 (14-26) | 0.98 |
| PVR, mmHg | 79/134 | 3.0 (1.7-4.3) | 3.2 (2.1-4.0) | 2.8 (1.5-5.0) | 0.73 |
| LVEF, % | 131/134 | 55 (45-62) | 55 (46-62) | 53 (45-60) | 0.49 |
| TAPSE, mm | 116/134 | 17 (14-19) | 18 (14-20) | 17 (14-18) | 0.49 |
| RV basal diameter, mm | 130/134 | 46.5 ± 7.9 | 44.9 ± 7.7 | 48.7 ± 7.7 | 0.007 |
| RV-FAC, % | 131/134 | 39.4 ± 10.6 | 40.4 ± 11.1 | 38.1 ± 9.8 | 0.23 |
| Degenerative TR, n (%) | 103/134 | 4 (3.9) | 2 (3.3) | 2 (4.7) | >0.99 |
| Atrial TR, n (%) | 103/134 | 53 (51.5) | 39 (65.0) | 14 (32.6) | 0.001 |
| Ventricular TR, n (%) | 103/134 | 46 (44.7) | 19 (31.7) | 27 (62.8) | 0.028 |
| Severe TR, n (%) | 134/134 | 60 (44.8) | 47 (63.5) | 13 (21.7) | <0.001 |
| Massive TR, n (%) | 134/134 | 48 (35.2) | 24 (32.4) | 24 (40.0) | 0.37 |
| Torrential TR, n (%) | 134/134 | 26 (19.4) | 3 (4.1) | 23 (38.3) | <0.001 |
| Mild TR achieved, n (%) | 134/134 | 61 (45.5) | 54 (73.0) | 7 (11.7) | <0.001 |
| Procedure time, min | 55/134 | 88 (65-120) | 79 (62-105) | 115 (85-148) | 0.022 |
| Number of implanted devices, n | 134/134 | 2 (1-2) | 2 (1-2) | 2 (1-2) | 0.65 |
| Technical success, n (%) | 103/134 | 93 (90.3) | 58 (96.7) | 35 (81.4) | 0.015 |
| Device success, n (%) | 103/134 | 67 (65.1) | 51 (85) | 16 (37.2) | <0.001 |
| All-cause mortality at 30 days, n (%) | 103/134 | 5 (4.9) | 1 (1.7) | 4 (9.3) | 0.17 |
| Cardiovascular mortality at 30 days, n (%) | 103/134 | 4 (3.9) | 1 (1.7) | 3 (7.0) | 0.33 |
| Bleeding requiring transfusion, n (%) | 103/134 | 6 (5.8) | 3 (5.0) | 3 (7.0) | >0.99 |
| Renal failure requiring dialysis, n (%) | 103/134 | 5 (4.9) | 4 (6.7) | 1 (2.3) | 0.38 |
| Device detatchment, n (%) | 103/134 | 6 (5.8) | 3 (5.0) | 3 (7.0) | >0.99 |
| Reintervention for tricuspid valve dysfunction at 30 days, n (%) | 103/134 | 3 (2.9) | 2 (3.3) | 1 (2.3) | >0.99 |
| Cardiac structural complications, n (%) | 103/134 | 1 (1.0) | 1 (1.7) | 0 (0.0) | >0.99 |
| Cerebrovascular event at 30 days, n (%) | 103/134 | 1 (1.0) | 1 (1.7) | 0 (0.0) | >0.99 |

TR = Tricuspid Regurgitation, BMI = Body-Mass-Index, NYHA-FC = New York Heart Association Functional Class, COPD = Chronic Obstructive Pulmonary Disease, CAD = Coronary Artery Disease, mPAP = Mean Pulmonary Artery Pressure, PCWP = Pulmonary Capillary Wedge Pressure, PVR = Pulmonary Vascular Resistance, LVEF = Left-ventricular Ejection Fraction, TAPSE = Tricuspid Annular Systolic Excursion, RV = Right Ventricle, RV-FAC = Right-ventricular Fractional Area Change.

**Supplemental Table 4:** Clinical and Procedural Details Based on GLIDE Score for Patients Treated with the Pascal System.

|  |  | **All** | **GLIDE Score**  **0 - 1** | **GLIDE Score**  **≥ 2** |  |
| --- | --- | --- | --- | --- | --- |
|  | Availability | *n* = 202 | *n* = 120 | *n* = 82 | *P*-value |
| Age, years | 202 / 202 | 81 (77-84) | 81 (77-83) | 81 (76-84) | 0.68 |
| Male, n (%) | 202 / 202 | 88 (43.6) | 53 (44.2) | 35 (42.7) | 0.89 |
| BMI, kg/m^2^ | 202 / 202 | 25.2 (22.8-28.7) | 25.0 (22.2-28.8) | 25.5 (23.4-28.7) | 0.29 |
| EuroScore II, % | 202 / 202 | 4.8 (3.0-9.0) | 4.6 (3.1-8.4) | 5.2 (3.0-9.2) | 0.71 |
| NYHA-FC ≥ III, n (%) | 158 / 202 | 78 (49.4) | 44 (44.0) | 34 (58.6) | 0.56 |
| Atrial Fibrillation, n (%) | 202 / 202 | 181 (90.1) | 109 (90.8) | 72 (88.9) | 0.49 |
| Diabetes, n (%) | 202 / 202 | 50 (24.8) | 32 (26.7) | 18 (22.0) | 0.51 |
| COPD, n (%) | 202 / 202 | 33 (16.3) | 16 (13.3) | 17 (20.7) | 0.18 |
| CAD, n (%) | 202 / 202 | 89 (44.1) | 55 (45.8) | 34 (41.5) | 0.57 |
| History of Cardiac  Surgery, n (%) | 202 / 202 | 57 (28.2) | 29 (24.2) | 28 (34.2) | 0.15 |
| History of Stroke, n (%) | 191 / 202 | 27 (14.1) | 14 (12.3) | 13 (16.9) | 0.41 |
| Renal failure  requiring dialysis, n (%) | 202 / 202 | 5 (2.5) | 3 (2.5) | 2 (2.4) | >0.99 |
| NT-proBNP, pg/ml | 182 / 202 | 2508 (1465-4844) | 2350 (1400-4110) | 2782 (1728-6080) | 0.09 |
| mPAP, mmHg | 153 / 202 | 28 (23-34) | 28 (22-33) | 28 (26-37) | 0.19 |
| PCWP, mmHg | 146 / 202 | 18 (14-22) | 18 (13-22) | 20 (16-25) | 0.036 |
| PVR, mmHg | 132 / 202 | 3.0 (2.0-4.0) | 3.1 (2.0-4-0) | 2.7 (1.9-4.0) | 0.43 |
| LVEF, % | 200 / 202 | 57 (50-63) | 57 (50-63) | 55 (51-65) | 0.92 |
| TAPSE, mm | 181 / 202 | 17 (14-20) | 17 (14-20) | 16 (14-19) | 0.57 |
| RV basal diameter, mm | 196 / 202 | 46 (42-53) | 46 (41-50) | 48 (42-54) | 0.019 |
| RV-FAC, % | 195 / 202 | 40.9 ± 11.2 | 41.1 ± 11.5 | 40.6 ± 10.9 | 0.75 |
| Degenerative TR, n (%) | 191 / 202 | 8 (4.2) | 5 (4.4) | 3 (3.9) | >0.99 |
| Atrial TR, n (%) | 191 / 202 | 85 (44.5) | 50 (43.9) | 35 (45.5) | 0.89 |
| Ventricular TR, n (%) | 191 / 202 | 98 (51.3) | 59 (51.8) | 39 (50.7) | 0.89 |
| Severe TR, n (%) | 202 / 202 | 104 (51.5) | 86 (71.7) | 18 (22.0) | >0.99 |
| Massive TR, n (%) | 202 / 202 | 67 (33.2) | 31 (25.8) | 36 (43.9) | 0.01 |
| Torrential TR, n (%) | 202 / 202 | 31 (15.4) | 3 (2.5) | 28 (34.2) | >0.99 |
| Mild TR achieved, n (%) | 202 / 202 | 103 (51.0) | 91 (75.8) | 12 (14.6) | >0.99 |
| Procedure time, min | 133 / 202 | 107 (73-135) | 91 (67-120) | 129 (93-166) | <0.001 |
| Number of implanted devices, n | 202 / 202 | 2 (1-2) | 2 (1-2) | 2 (2-2) | 0.004 |
| Technical success, n (%) | 191 / 202 | 185 (96.9) | 113 (99.1) | 72 (93.5) | 0.13 |
| Device success, n (%) | 191 / 202 | 136 (71.2) | 100 (87.7) | 36 (46.8) | <0.001 |
| All-cause mortality at 30 days, n (%) | 190 / 202 | 3 (1.6) | 2 (1.8) | 1 (1.3) | >0.99 |
| Cardiovascular mortality at 30 days, n (%) | 190 / 202 | 3 (1.6) | 2 (1.8) | 1 (1.3) | >0.99 |
| Bleeding requiring transfusion, n (%) | 191 / 202 | 9 (4.7) | 4 (3.5) | 5 (6.5) | 0.49 |
| Renal failure requiring dialysis, n (%) | 191 / 202 | 1 (0.5) | 1 (0.9) | 0 (0.0) | >0.99 |
| Device detatchment, n (%) | 191 / 202 | 5 (2.6) | 1 (0.9) | 4 (5.2) | 0.16 |
| Reintervention for tricuspid valve dysfunction at 30 days, n (%) | 189 / 202 | 3 (1.6) | 1 (0.9) | 2 (2.6) | 0.57 |
| Cardiac structural complications, n (%) | 191 / 202 | 1 (0.5) | 0 (0.0) | 1 (1.3) | 0.41 |
| Cerebrovascular event at 30 days, n (%) | 189 / 202 | 0 (0.0) | 0 (0.0) | 0 (0.0) | >0.99 |

TR = Tricuspid Regurgitation, BMI = Body-Mass-Index, NYHA-FC = New York Heart Association Functional Class, COPD = Chronic Obstructive Pulmonary Disease, CAD = Coronary Artery Disease, mPAP = Mean Pulmonary Artery Pressure, PCWP = Pulmonary Capillary Wedge Pressure, PVR = Pulmonary Vascular Resistance, LVEF = Left-ventricular Ejection Fraction, TAPSE = Tricuspid Annular Systolic Excursion, RV = Right Ventricle, RV-FAC = Right-ventricular Fractional Area Change.

**Supplemental Table 5:** Predictive Performance of the GLIDE Score Based on Implanted Device System.

|  | **TriClip** | **Pascal** |
| --- | --- | --- |
| Area under the curve | 0.85 (95% CI: 0.79 - 0.91)* | 0.88 (95% CI: 0.84 - 0.92)* |
| Sensitivity | 0.89 (95% CI: 0.78 - 0.94) | 0.88 (95% CI: 0.81 - 0.93) |
| Specificity | 0.73 (95% CI: 0.61 - 0.82) | 0.71 (95% CI: 0.61 - 0.79) |
| Positive predictive value | 0.73 (95% CI: 0.62 - 0.82) | 0.76 (95% CI: 0.68 - 0.83) |
| Negative predictive value | 0.88 (95% CI: 0.78 - 0.94) | 0.85 (95% CI: 0.76 - 0.91) |

CI = confidence interval. *DeLong test revealed no significant differences between the area under the curve for both devices (*P* = 0.45).

**Supplemental Table 6:** Clinical and Procedural Details Based on the Presence of Torrential TR.

|  |  | **All** | **Non-torrential TR** | **Torrential TR** |  |
| --- | --- | --- | --- | --- | --- |
|  | Availability | *n* = 336 | *n* = 279 | *n* = 57 | *P*-value |
| Age, years | 336/336 | 81 (77-84) | 81 (77-84) | 81 (76-85) | 0.81 |
| Male, n (%) | 336/336 | 150 (44.6) | 116 (41.6) | 34 (59.7) | 0.013 |
| BMI, kg/m^2^ | 336/336 | 25.2 (22.7-28.4) | 25.4 (22.6-28.7) | 24.1 (22.8-27.5) | 0.23 |
| EuroScore II, % | 336/336 | 5.2 (3.3-9.1) | 5.2 (3.3-8.9) | 5.0 (3.3-10.4) | 0.84 |
| NYHA-FC ≥ III, n (%) | 261/336 | 146 (55.9) | 117 (54.7) | 29 (61.7) | 0.24 |
| Atrial Fibrillation, n (%) | 336/336 | 306 (91.3) | 254 (91.4) | 52 (91.2) | >0.99 |
| Diabetes, n (%) | 336/336 | 80 (23.8) | 69 (24.7) | 11 (19.3) | 0.50 |
| COPD, n (%) | 336/336 | 57 (17.0) | 47 (16.9) | 10 (17.5) | 0.85 |
| CAD, n (%) | 336/336 | 161 (47.9) | 132 (47.3) | 29 (50.9) | 0.66 |
| History of Cardiac  Surgery, n (%) | 336/336 | 102 (30.4) | 82 (29.4) | 20 (35.1) | 0.43 |
| History of Stroke, n (%) | 293/336 | 44 (15.0) | 40 (15.9) | 4 (9.8) | 0.19 |
| Renal failure  requiring dialysis, n (%) | 336/336 | 12 (3.6) | 7 (2.5) | 5 (8.8) | 0.036 |
| NT-proBNP, pg/ml | 306/336 | 2695 (1427-5228) | 2710 (1427-4957) | 2686 (1440-6279) | 0.57 |
| mPAP, mmHg | 263/336 | 28 (23-35) | 28 (23-35) | 29 (24-33) | 0.99 |
| PCWP, mmHg | 250/336 | 19 (14-24) | 19 (14-23) | 19 (16-25) | 0.23 |
| PVR, mmHg | 211/336 | 3 (1.9-4.1) | 3.2 (1.9-4.3) | 2.3 (1.5-3.1) | 0.013 |
| LVEF, % | 331/336 | 55 (48-63) | 55 (50-63) | 55 (46-63) | 0.36 |
| TAPSE, mm | 297/336 | 17 (14-20) | 17 (14-20) | 16 (13-18) | 0.07 |
| RV basal diameter, mm | 326/336 | 46 (41-53) | 45 (41-50) | 53 (46-58) | <0.001 |
| RV-FAC, % | 326/336 | 40.3 ± 11.0 | 40.7 ± 11.2 | 38.2 ± 9.5 | 0.10 |
| Degenerative TR, n (%) | 294/336 | 12 (4.1) | 11 (4.4) | 1 (2.4) | 0.70 |
| Atrial TR, n (%) | 294/336 | 138 (46.9) | 125 (49.4) | 13 (31.7) | 0.002 |
| Ventricular TR, n (%) | 294/336 | 144 (49.0) | 117 (46.3) | 27 (65.9) | 0.47 |
| GLIDE Score sum | 336/336 | 1 (0-3) | 1 (0-2) | 3 (3-4) | <0.001 |
| GLIDE Score 0-1, n (%) | 336/336 | 194 (57.7) | 188 (67.4) | 6 (10.5) | <0.001 |
| Mild TR achieved, n (%) | 336 / 336 | 164 (48.8) | 161 (57.7) | 3 (5.3) | <0.001 |
| Procedure time, min | 188/336 | 101 (71-132) | 94 (69-123) | 153 (133-196) | <0.001 |
| Number of implanted devices, n | 336/336 | 2 (1-2) | 2 (1-2) | 2 (1-2) | 0.13 |
| Technical success, n (%) | 294/336 | 278 (94.6) | 239 (94.5) | 39 (95.1) | 0.003 |
| Device success, n (%) | 294/336 | 203 (69.1) | 186 (73.5) | 17 (41.5) | <0.001 |
| All-cause mortality at 30 days, n (%) | 293/336 | 8 (2.7) | 8 (3.2) | 0 (0.0) | 0.36 |
| Cardiovascular mortality at 30 days, n (%) | 293/336 | 7 (2.4) | 7 (2.8) | 0 (0.0) | 0.61 |
| Bleeding requiring transfusion, n (%) | 294/336 | 15 (5.1) | 12 (4.7) | 3 (7.3) | 0.73 |
| Renal failure requiring dialysis, n (%) | 294/336 | 6 (2.0) | 6 (2.4) | 0 (0.0) | 0.60 |
| Device detatchment, n (%) | 294/336 | 11 (3.7) | 8 (3.2) | 3 (7.3) | 0.41 |
| Reintervention for tricuspid valve dysfunction at 30 days, n (%) | 292/336 | 6 (2.1) | 4 (1.6) | 2 (4.9) | 0.27 |
| Cardiac structural complications, n (%) | 294/336 | 2 (0.7) | 1 (0.4) | 1 (2.4) | 0.31 |
| Cerebrovascular event at 30 days, n (%) | 292/336 | 1 (0.3) | 1 (0.4) | 0 (0.0) | >0.99 |

TR = Tricuspid Regurgitation, BMI = Body-Mass-Index, NYHA-FC = New York Heart Association Functional Class, COPD = Chronic Obstructive Pulmonary Disease, CAD = Coronary Artery Disease, mPAP = Mean Pulmonary Artery Pressure, PCWP = Pulmonary Capillary Wedge Pressure, PVR = Pulmonary Vascular Resistance, LVEF = Left-ventricular Ejection Fraction, TAPSE = Tricuspid Annular Systolic Excursion, RV = Right Ventricle, RV-FAC = Right-ventricular Fractional Area Change.
